# Supplementary material for: ROS amplification drives mouse spermatogonial stem cell self-renewal
Source: Life Sci Alliance. 2019 Apr 2;2(2):e201900374. doi: 10.26508/lsa.201900374 (PMC6448598; doi:10.26508/lsa.201900374)
Supplement: Supplementary file 1 [file LSA-2019-00374_TableS1.docx]

**Table S1 : Statistical analysis of ROS levels**

| Figure | Treatment | p-values | Control |
| --- | --- | --- | --- |
| Fig. 1D | VX-745 | <0.0001 | Culture medium + DMSO |
| Fig. 1G | *Mapka14* OE | <0.0001 | Control (*Eyfp*) OE |
| Fig. 2F | *Mapka14*^f/f^ GS + AxCANCre | <0.0001 | *Mapk14* ^f/f^ GS +AxCANLacZ |
| Fig. 3E | *Map2k5* OE | <0.0001 | Control (*Eyfp*) OE |
| Fig. 4F | *Mapka7*^f/f^ GS + AxCANCre | <0.0001 | *Mapk7* ^f/f^ GS + AxCANLacZ |
| Fig. 5E | *Nox1* OE | <0.0001 | Control (*Eyfp*) OE |
| Fig. 5G | *Noxa2* KD | <0.0001 | Scramble shRNA |
| Fig. 5I | *Noxa2* OE | <0.0001 | Control (*Eyfp*) OE |
| Fig. 5J | *Noxa2* KD + *Nox1* OE | <0.0001 | *Noxa2* KD + *Eyfp* OE |
| Fig. 6A | *Bcl6b* KD | <0.0001 | Scramble shRNA |
| Fig. 6A | *Sohlh1* KD | 0.0095 | Scramble shRNA |
| Fig. 6B | *Map2k5* OE + *Bcl6b* KD | <0.0001 | *Map2k5* OE + Scramble shRNA |
| Fig. 6B | *Bcl6b* OE | <0.0001 | Control (*Eyfp*) OE |
| Fig. 7C | *Etv5* KD | <0.0001 | Scramble shRNA |
| Fig. 7C | *Etv5* OE | <0.0001 | Control (*Eyfp*) OE |
| Fig. 7C | *Map2k5* OE + *Etv5* KD | <0.0001 | *Map2k5* OE + Scramble shRNA |
| Fig. 7C | NF + *Bcl6b* OE | <0.0001 | Control (*Eyfp*) OE |
| Fig. S1A | NF + FG | <0.0001 | Culture medium (NF) |
| Fig. S1B | *Nox1* KD | <0.0001 | Scramble shRNA |
| Fig. S1C | NF + *Map2k5* OE | <0.0001 | Control (*Eyfp*) OE |
| Fig. S1D | *Map2k5* OE + *Nox1* KD | <0.0001 | *Map2k5* OE + Scramble shRNA |

Results of three experiments. Analyzed by linear regression.
