# Supplementary material for: ROS amplification drives mouse spermatogonial stem cell self-renewal
Source: Life Sci Alliance. 2019 Apr 2;2(2):e201900374. doi: 10.26508/lsa.201900374 (PMC6448598; doi:10.26508/lsa.201900374)
Supplement: Supplementary file 2 [file LSA-2019-00374_TableS2.docx]

**Table S2 : Quantification of Western blot analysis**

| Figure | Treatment | Antigen | Fold change  Mean ± SEM | p-values | Control |
| --- | --- | --- | --- | --- | --- |
| Fig. 1E | H_2_O_2_ | p-MAPK14 | 1.758±0.082 | 0.01350 | Culture medium |
| Fig. 1F | NAC | p-MAPK14 | 0.438±0.032 | 0.00077 | Culture medium + DMSO |
| Fig. 3C | Starve+FGF2, GDNF | p-MAPK7 | 5.349±0.393 | 0.00095 | Starved (-FGFG2, -GDNF) |
| Fig. 3F | LPA | p-MAPK7 | 0.364±0.060 | 0.00663 | Culture medium + DMSO |
| Fig. 3F | DPI | p-MAPK7 | 0.118±0.048 | 0.00324 | Culture medium + DMSO |
| Fig. 3F | Apocynin | p-MAPK7 | 0.268±0.141 | 0.01220 | Culture medium + DMSO |
| Fig. 3G | H_2_O_2_ （4 h） | p-MAPK7 | 1.134±0.190 | 0.66770 | Culture medium |
| Fig. 3G | H_2_O_2_ （8 h） | p-MAPK7 | 4.907±0.720 | 0.01219 | Culture medium |
| Fig. 3H | *Map2k5* OE | p-MAPK7 | 4.872±1.041 | 0.02558 | Control (*Eyfp*) OE |
| Fig. 3H | *Map2k5* OE | p-MAPK14 | 0.903±0.415 | 0.82680 | Control (*Eyfp*) OE |
| Fig. 3H | *Mapk14* OE | p-MAPK7 | 2.373±0.092 | 0.00494 | Control (*Eyfp*) OE |
| Fig. 3H | *Mapk14* OE | p-MAPK14 | 2.085±0.084 | 0.00008 | Control (*Eyfp*) OE |
| Fig. 3H | *Mapk14* OE | p- MAP2K5 | 2.618±0.074 | 0.00004 | Control (*Eyfp*) OE |
| Fig. 3I | SB203589 | p-MAPK7 | 0.119±0.045 | 0.00027 | Culture medium + DMSO |
| Fig. 3I | SB203589 | p-MAPK14 | 0.243±0.082 | 0.00426 | Culture medium + DMSO |
| Fig. 3I | XMD 8-92 | p-MAPK7 | 0.08±0.003 | 0.00278 | Culture medium + DMSO |
| Fig. 3I | XMD 8-92 | p-MAPK14 | 0.895±0.018 | 0.48766 | Culture medium + DMSO |
| Fig. 3J | AxCANCre (*Mapk14*) | p-MAPK7 | 0.378±0.114 | 0.00769 | AcCANLacZ |
| Fig. 3J | AxCANCre (*Mapk14*) | p-MAPK14 | 0.186±0.094 | 0.03164 | AcCANLacZ |
| Fig. 4H | AxCANCre (*Mapk7*) | p-MAPK7 | 0.557±0.111 | 0.03389 | AcCANLacZ |
| Fig. 4H | AxCANCre (*Mapk7*) | p-MAPK14 | 1.162±0.160 | 0.67408 | AcCANLacZ |
| Fig. 7E | *Mapk2k5* OE (2 days) | p-MAPK14 | 0.987±0.037 | 0.76883 | Control (*Eyfp*) OE |
| Fig. 7E | *Mapk2k5* OE (5 days) | p-MAPK14 | 1.560±0.079 | 0.00473 | Control (*Eyfp*) OE |
| Fig. 7E | *Mapk2k5* OE (6 days) | p-MAPK14 | 1.611±0.013 | 0.00077 | Control (*Eyfp*) OE |
| Fig. 7E | *Mapk2k5* OE (7 days) | p-MAPK14 | 1.016±0.017 | 0.73492 | Control (*Eyfp*) OE |
| Fig. 7F | *Bcl6b* KD | p-MAPK7 | 0.279±0.053 | 0.00346 | Scramble shRNA |
| Fig. 7F | *Bcl6b* KD | p-MAPK14 | 0. 346±0.077 | 0.00178 | Scramble shRNA |
| Fig. S5C | *Etv5* OE | BCL6B | 1.200±0.180 | 0.12164 | Control (*Eyfp*) OE |
| Fig. S5C | *Map2k5* OE | BCL6B | 0.865±0.076 | 0.19378 | Control (*Eyfp*) OE |
| Fig. S5C | *Nox1* OE | BCL6B | 0.964±0.138 | 0.42471 | Control (*Eyfp*) OE |
| Fig. S5C | H_2_O_2_ | BCL6B | 0.952±0.024 | 0.65242 | Culture medium |
| Fig. S5C | AxCANCre (*Mapk7*) | BCL6B | 0.970±0.004 | 0.86270 | AcCANLacZ |
| Fig. S5C | AxCANCre (*Mapk14*) | BCL6B | 1.003±0.154 | 0.89873 | AcCANLacZ |

Relative expression levels were compared with control ACTB levels. Results of three experiments.
