# Supplementary material for: ROS amplification drives mouse spermatogonial stem cell self-renewal
Source: Life Sci Alliance. 2019 Apr 2;2(2):e201900374. doi: 10.26508/lsa.201900374 (PMC6448598; doi:10.26508/lsa.201900374)
Supplement: Supplementary file 3 [file LSA-2019-00374_TableS3.docx]

**Table S3 : Microarray analysis**

**Top 50 downregulated genes in *Nox1* KD**

| Fold  change | Gene symbol | Gene description |
| --- | --- | --- |
| -3.34 | Rnf213 | ring finger protein 213 |
| -2.58 | Tgtp1 | T-cell specific GTPase 1 |
| -2.19 | Mx2 | myxovirus (influenza virus) resistance 2 |
| -2.16 | Herc6 | hect domain and RLD 6 |
| -2.09 | Ddx60 | DEAD (Asp-Glu-Ala-Asp) box polypeptide 60 |
| -2.07 | Psmb8 | proteasome (prosome, macropain) subunit, beta type 8 (large multifunctional peptidase 7) |
| -2.07 | Gm12185 | predicted gene 12185 |
| -1.99 | Gbp6 | guanylate binding protein 6 |
| -1.96 | Defb47 | defensin beta 47 |
| -1.95 | Trim14 | tripartite motif-containing 14 |
| -1.92 | 4922501L14Rik | RIKEN cDNA 4922501L14 gene |
| -1.91 | Oas1a | 2'-5' oligoadenylate synthetase 1A |
| -1.90 | Irgm2 | immunity-related GTPase family M member 2 |
| -1.89 | Epsti1 | epithelial stromal interaction 1 (breast) |
| -1.88 | Tap1 | transporter 1, ATP-binding cassette, sub-family B (MDR/TAP) |
| -1.88 | Ifi44 | interferon-induced protein 44 |
| -1.84 | Gdap10 | ganglioside-induced differentiation-associated-protein 10 |
| -1.84 | H2-T22 | histocompatibility 2, T region locus 22 |
| -1.83 | Gm1527 | predicted gene 1527 |
| -1.82 | Oas1g | 2'-5' oligoadenylate synthetase 1G |
| -1.80 | Isg20 | interferon-stimulated protein |
| -1.78 | Snap25 | synaptosomal-associated protein 25 |
| -1.77 | 2010002M12Rik | RIKEN cDNA 2010002M12 gene |
| -1.77 | Oas1c | 2'-5' oligoadenylate synthetase 1C |
| -1.76 | Gbp11 | guanylate binding protein 11 |
| -1.76 | Trim21 | tripartite motif-containing 21 |
| -1.76 | Cdc26 | cell division cycle 26 |
| -1.75 | D630029K05Rik | RIKEN cDNA D630029K05 gene |
| -1.73 | D130062J21Rik | RIKEN cDNA D130062J21 gene |
| -1.73 | Tcfap2a | transcription factor AP-2, alpha |
| -1.71 | Ftl1 | ferritin light chain 1 |
| -1.70 | Slc35f3 | solute carrier family 35, member F3 |
| -1.70 | Scarna8 | small Cajal body-specific RNA 8 |
| -1.69 | Zbp1 | Z-DNA binding protein 1 |
| -1.69 | Dnahc5 | dynein, axonemal, heavy chain 5 |
| -1.68 | Dhx58 | DEXH (Asp-Glu-X-His) box polypeptide 58 |
| -1.66 | Gbp3 | guanylate binding protein 3 |
| -1.65 | Mpa2l | macrophage activation 2 like |
| -1.65 | A230050P20Rik | RIKEN cDNA A230050P20 gene |
| -1.64 | Gm1524 | predicted gene 1524 |
| -1.63 | Ctag2 | cancer/testis antigen 2 |
| -1.63 | Usp18 | ubiquitin specific peptidase 18 |
| -1.63 | Ms4a4d | membrane-spanning 4-domains, subfamily A, member 4D |
| -1.63 | Sp100 |  |
| -1.62 | Eif2ak2 | eukaryotic translation initiation factor 2-alpha kinase 2 |
| -1.62 | Ube2l6 | ubiquitin-conjugating enzyme E2L 6 |
| -1.62 | Gm5431 | predicted gene 5431 |
| -1.62 | Peg10 | paternally expressed 10 |
| -1.62 | Stat2 | signal transducer and activator of transcription 2 |
| -1.61 | Mx1 | myxovirus (influenza virus) resistance 1 |

**Top 50 upregulated genes in *Nox1* KD**

| Fold  change | Gene symbol | Gene description |
| --- | --- | --- |
| 3.63 | Cxcl2 | chemokine (C-X-C motif) ligand 2 |
| 2.78 | Fcer1g | Fc receptor, IgE, high affinity I, gamma polypeptide |
| 2.61 | Lphn2 | latrophilin 2 |
| 2.43 | Acta2 | actin, alpha 2, smooth muscle, aorta |
| 2.33 | Gm10785 | predicted gene 10785 |
| 2.31 | Csn3 | casein kappa |
| 2.22 | Itpripl2 | inositol 1,4,5-triphosphate receptor interacting protein-like 2 |
| 2.19 | Anxa5 | annexin A5 |
| 2.19 | Gm10772 | predicted gene 10772 |
| 2.18 | Zfand2a | zinc finger, AN1-type domain 2A |
| 2.18 | Mpzl2 | myelin protein zero-like 2 |
| 2.15 | Olr1 | oxidized low density lipoprotein (lectin-like) receptor 1 |
| 2.13 | Il6 | interleukin 6 |
| 2.13 | Cxcl5 | chemokine (C-X-C motif) ligand 5 |
| 2.06 | Gm10857 | predicted gene 10857 |
| 2.05 | Cd59a | CD59a antigen |
| 2.04 | Gm5458 | predicted gene 5458 |
| 2.03 | BC013529 |  |
| 1.99 | Adh6b | alcohol dehydrogenase 6B (class V) |
| 1.97 | Gm3002 | alpha-takusan pseudogene |
| 1.96 | Fam96a | family with sequence similarity 96, member A |
| 1.95 | Lox | lysyl oxidase |
| 1.95 | Tnfrsf23 | tumor necrosis factor receptor superfamily, member 23 |
| 1.95 | Ccl7 | chemokine (C-C motif) ligand 7 |
| 1.93 | Tsen15 | tRNA splicing endonuclease 15 homolog (S. cerevisiae) |
| 1.93 | Cd80 | CD80 antigen |
| 1.91 | Ube2w | ubiquitin-conjugating enzyme E2W (putative) |
| 1.90 | Unc13c | unc-13 homolog C (C. elegans) |
| 1.90 | Cenpq | centromere protein Q |
| 1.89 | Fah | fumarylacetoacetate hydrolase |
| 1.88 | AI607873 |  |
| 1.87 | Rpa3 | replication protein A3 |
| 1.86 | Tagln | transgelin |
| 1.86 | Phf20 | PHD finger protein 20 |
| 1.84 | Cript | cysteine-rich PDZ-binding protein |
| 1.84 | A630089N07Rik | RIKEN cDNA A630089N07 gene |
| 1.82 | 1700084J12Rik | RIKEN cDNA 1700084J12 gene |
| 1.82 | Csf1 | colony stimulating factor 1 (macrophage) |
| 1.82 | Arhgap42 | Rho GTPase activating protein 42 |
| 1.81 | Sumo1 | SMT3 suppressor of mif two 3 homolog 1 (yeast) |
| 1.80 | Gm13242 | predicted gene 13242 |
| 1.80 | Tbc1d7 | TBC1 domain family, member 7 |
| 1.79 | Ccbl2 | cysteine conjugate-beta lyase 2 |
| 1.79 | Rex2 | reduced expression 2 |
| 1.79 | Uprt | uracil phosphoribosyltransferase (FUR1) homolog (S. cerevisiae) |
| 1.79 | Prl2c3 | prolactin family 2, subfamily c, member 3 |
| 1.77 | Aspn | asporin |
| 1.76 | Gpx3 | glutathione peroxidase 3 |
| 1.76 | Ccdc80 | coiled-coil domain containing 80 |
| 1.76 | Slc25a5 | solute carrier family 25 (mitochondrial carrier, adenine nucleotide translocator), member 5 |

**Top 50 downregulated genes in *Mapk7* KO**

| Fold  change | Gene symbol | Gene description |
| --- | --- | --- |
| -4.81 | Ifi205 | interferon activated gene 205 |
| -2.90 | Ccl11 | chemokine (C-C motif) ligand 11 |
| -2.86 | Ssty2 | spermiogenesis specific transcript on the Y 2 |
| -2.82 | Lum | lumican |
| -2.71 | Ly6a | lymphocyte antigen 6 complex, locus A |
| -2.67 | Dcn | decorin |
| -2.58 | LOC100042196 | y-linked testis-specific protein 1-like |
| -2.55 | LOC100039753 | spermiogenesis specific transcript on the Y family member |
| -2.54 | LOC665746 | y-linked testis-specific protein 1-like |
| -2.53 | LOC100040223 | similar to spermiogenesis specific transcript on the Y 1 |
| -2.51 | LOC100039552 | y-linked testis-specific protein 1-like |
| -2.49 | LOC665406 | y-linked testis-specific protein 1-like |
| -2.47 | Ssty1 | spermiogenesis specific transcript on the Y 1 |
| -2.46 | LOC100041704 | y-linked testis-specific protein 1-like |
| -2.36 | LOC100041256 | hypothetical protein LOC100041256 |
| -2.34 | LOC100042359 | y-linked testis-specific protein 1-like |
| -2.25 | Igsf10 | immunoglobulin superfamily, member 10 |
| -2.25 | Steap4 | STEAP family member 4 |
| -2.24 | LOC100039147 | y-linked testis-specific protein 1-like |
| -2.24 | LOC100040031 | hypothetical protein LOC100040031 |
| -2.20 | Itm2a | integral membrane protein 2A |
| -2.20 | Ly6c1 | lymphocyte antigen 6 complex, locus C1 |
| -2.19 | LOC100040235 | y-linked testis-specific protein 1-like |
| -2.16 | LOC665128 | y-linked testis-specific protein 1-like |
| -2.15 | Igsf10 | immunoglobulin superfamily, member 10 |
| -2.08 | Rfc4 | replication factor C (activator 1) 4 |
| -2.08 | LOC380994 | similar to Sycp3 like Y-linked |
| -2.08 | LOC665698 | y-linked testis-specific protein 1-like |
| -2.06 | Saa3 | serum amyloid A 3 |
| -2.05 | F3 | coagulation factor III |
| -2.04 | Sly | Sycp3 like Y-linked |
| -1.98 | Vmn2r43 | vomeronasal 2, receptor 43 |
| -1.95 | Lss | lanosterol synthase |
| -1.95 | LOC380994 | similar to Sycp3 like Y-linked |
| -1.93 | Snord116 | small nucleolar RNA, C/D box 116 cluster |
| -1.92 | Egr2 | early growth response 2 |
| -1.91 | Sc4mol | sterol-C4-methyl oxidase-like |
| -1.91 | Serpina3n | serine (or cysteine) peptidase inhibitor, clade A, member 3N |
| -1.90 | Ptx3 | pentraxin related gene |
| -1.89 | Tgfbi | transforming growth factor, beta induced |
| -1.86 | Snord115 | Small nucleolar RNA, C/D Box 115 cluster |
| -1.86 | Igfbp3 | insulin-like growth factor binding protein 3 |
| -1.85 | Mmp13 | matrix metallopeptidase 13 |
| -1.84 | Thbs2 | thrombospondin 2 |
| -1.83 | Snord37 | small nucleolar RNA, C/D box 37 |
| -1.78 | Gm1943 | WD repeat domain 70 pseudogene |
| -1.78 | Vcan | versican |
| -1.77 | Mmp3 | matrix metallopeptidase 3 |
| -1.75 | Postn | periostin, osteoblast specific factor |
| -1.74 | Idi1 | isopentenyl-diphosphate delta isomerase |

**Top 50 upregulated genes in *Mapk7* KO**

| Fold  change | Gene symbol | Gene description |
| --- | --- | --- |
| 4.25 | Crct1 | cysteine-rich C-terminal 1 |
| 2.42 | Lce1h | late cornified envelope 1H |
| 2.22 | Gsta4 | glutathione S-transferase, alpha 4 |
| 2.19 | Snord118 | small nucleolar RNA, C/D box 118 |
| 2.15 | Mustn1 | musculoskeletal, embryonic nuclear protein 1 |
| 2.13 | Tslp | thymic stromal lymphopoietin |
| 2.07 | Sema7a | sema domain, immunoglobulin domain (Ig), and GPI membrane anchor, (semaphorin) 7A |
| 1.97 | Fxyd5 | FXYD domain-containing ion transport regulator 5 |
| 1.95 | Stra8 | stimulated by retinoic acid gene 8 |
| 1.95 | Prl2c3 | prolactin family 2, subfamily c, member 3 |
| 1.92 | Timp1 | tissue inhibitor of metalloproteinase 1 |
| 1.82 | Cma2 | chymase 2, mast cell |
| 1.78 | Cryab | crystallin, alpha B |
| 1.77 | Slain2 | SLAIN motif family, member 2 |
| 1.77 | Crabp2 | cellular retinoic acid binding protein II |
| 1.76 | Stmn2 | stathmin-like 2 |
| 1.76 | Vbp1 | von Hippel-Lindau binding protein 1 |
| 1.75 | Serinc2 | serine incorporator 2 |
| 1.74 | Selm | selenoprotein M |
| 1.73 | Rhoc | ras homolog gene family, member C |
| 1.73 | Tagln | transgelin |
| 1.72 | Enpp1 | ectonucleotide pyrophosphatase/phosphodiesterase 1 |
| 1.71 | Alcam | activated leukocyte cell adhesion molecule |
| 1.70 | Slco2a1 | solute carrier organic anion transporter family, member 2a1 |
| 1.69 | S100a6 | S100 calcium binding protein A6 (calcyclin) |
| 1.69 | Mcpt9 | mast cell protease 9 |
| 1.68 | Ank | progressive ankylosis |
| 1.67 | Mmp10 | matrix metallopeptidase 10 |
| 1.66 | Crmp1 | collapsin response mediator protein 1 |
| 1.66 | Mcpt8 | mast cell protease 8 |
| 1.66 | Ccbe1 | collagen and calcium binding EGF domains 1 |
| 1.66 | Hamp2 | hepcidin antimicrobial peptide 2 |
| 1.65 | Grem1 | gremlin 1 |
| 1.65 | A530064D06Rik | RIKEN cDNA A530064D06 gene |
| 1.65 | Pcbd1 | pterin 4 alpha carbinolamine dehydratase/dimerization cofactor of hepatocyte nuclear factor 1 alpha (TCF1) 1 |
| 1.63 | Il33 | interleukin 33 |
| 1.63 | Raet1d | retinoic acid early transcript delta |
| 1.62 | Prg4 | proteoglycan 4 (megakaryocyte stimulating factor, articular superficial zone protein) |
| 1.61 | 1700013H16Rik | RIKEN cDNA 1700013H16 gene |
| 1.61 | Ttc9 | tetratricopeptide repeat domain 9 |
| 1.60 | Rnf7 | ring finger protein 7 |
| 1.60 | Fads3 | fatty acid desaturase 3 |
| 1.60 | Gal | galanin |
| 1.58 | 1700008I05Rik | RIKEN cDNA 1700008I05 gene |
| 1.57 | Runx3 | runt related transcription factor 3 |
| 1.57 | 9.Sep | septin 9 |
| 1.57 | Fgf2 | fibroblast growth factor 2 |
| 1.57 | Oaf | OAF homolog (Drosophila) |
| 1.56 | Cdh6 | cadherin 6 |
| 1.56 | Rhox4c | reproductive homeobox 4C |

**Top 50 downregulated genes in *Mapk14* KO**

| Fold  change | Gene symbol | Gene description |
| --- | --- | --- |
| -5.13 | Ednra | endothelin receptor type A |
| -4.49 | Inhba | inhibin beta-A |
| -4.11 | Prg4 | proteoglycan 4 (megakaryocyte stimulating factor, articular superficial zone protein) |
| -4.07 | Itga5 | integrin alpha 5 (fibronectin receptor alpha) |
| -3.92 | Mmp13 | matrix metallopeptidase 13 |
| -3.82 | Has2 | hyaluronan synthase 2 |
| -3.72 | Tfpi2 | tissue factor pathway inhibitor 2 |
| -3.71 | Prrx1 | paired related homeobox 1 |
| -3.62 | Anxa5 | annexin A5 |
| -3.62 | Tnfrsf23 | tumor necrosis factor receptor superfamily, member 23 |
| -3.61 | Stc1 | stanniocalcin 1 |
| -3.47 | Mmp10 | matrix metallopeptidase 10 |
| -3.43 | Cdh11 | cadherin 11 |
| -3.41 | Pdgfrb | platelet derived growth factor receptor, beta polypeptide |
| -3.40 | AI607873 |  |
| -3.40 | Ccl5 | chemokine (C-C motif) ligand 5 |
| -3.39 | Lgals3bp | lectin, galactoside-binding, soluble, 3 binding protein |
| -3.26 | Angptl2 | angiopoietin-like 2 |
| -3.15 | Hmga2 | high mobility group AT-hook 2 |
| -3.14 | Serpinb2 | serine (or cysteine) peptidase inhibitor, clade B, member 2 |
| -3.13 | Oaf | OAF homolog (Drosophila) |
| -3.09 | Ehd2 | EH-domain containing 2 |
| -3.08 | Acta2 | actin, alpha 2, smooth muscle, aorta |
| -3.07 | Pcdh7 | protocadherin 7 |
| -3.07 | S1pr3 | sphingosine-1-phosphate receptor 3 |
| -3.02 | Cd80 | CD80 antigen |
| -3.01 | Lox | lysyl oxidase |
| -2.94 | Ifi203 | interferon activated gene 203 |
| -2.93 | Olr1 | oxidized low density lipoprotein (lectin-like) receptor 1 |
| -2.90 | Cd97 | CD97 antigen |
| -2.88 | Ifi204 | interferon activated gene 204 |
| -2.88 | Ptges | prostaglandin E synthase |
| -2.81 | Gpx8 | glutathione peroxidase 8 (putative) |
| -2.80 | Antxr1 | anthrax toxin receptor 1 |
| -2.78 | Pi15 | peptidase inhibitor 15 |
| -2.78 | Ifitm3 | interferon induced transmembrane protein 3 |
| -2.77 | Ecm1 | extracellular matrix protein 1 |
| -2.77 | Ass1 | argininosuccinate synthetase 1 |
| -2.76 | Ly96 | lymphocyte antigen 96 |
| -2.75 | Grem1 | gremlin 1 |
| -2.74 | Cryab | crystallin, alpha B |
| -2.73 | Dab2 | disabled homolog 2 (Drosophila) |
| -2.71 | Ass1 | argininosuccinate synthetase 1 |
| -2.69 | Sema7a | sema domain, immunoglobulin domain (Ig), and GPI membrane anchor, (semaphorin) 7A |
| -2.68 | Penk | preproenkephalin |
| -2.67 | Igfbp7 | insulin-like growth factor binding protein 7 |
| -2.67 | Casp8 | caspase 8 |
| -2.66 | Fxyd5 | FXYD domain-containing ion transport regulator 5 |
| -2.66 | Cd109 | CD109 antigen |
| -2.65 | Cpe | carboxypeptidase E |

**Top 50 upregulated genes in *Mapk14* KO**

| Fold  change | Gene symbol | Gene description |
| --- | --- | --- |
| 3.88 | Fosb | FBJ osteosarcoma oncogene B |
| 2.78 | Cxcl2 | chemokine (C-X-C motif) ligand 2 |
| 2.00 | Rsph4a | radial spoke head 4 homolog A (Chlamydomonas) |
| 1.98 | Lyz2 | lysozyme 2 |
| 1.94 | Fos | FBJ osteosarcoma oncogene |
| 1.93 | Ldlr | low density lipoprotein receptor |
| 1.92 | Cd274 | CD274 antigen |
| 1.88 | Fbxo32 | F-box protein 32 |
| 1.88 | Cyp51 | cytochrome P450, family 51 |
| 1.88 | Snora44 | small nucleolar RNA, H/ACA box 44 |
| 1.86 | Atf3 | activating transcription factor 3 |
| 1.84 | Syne2 | synaptic nuclear envelope 2 |
| 1.83 | Egr1 | early growth response 1 |
| 1.79 | Sc4mol | sterol-C4-methyl oxidase-like |
| 1.77 | Idi1 | isopentenyl-diphosphate delta isomerase |
| 1.75 | Zfp36 | zinc finger protein 36 |
| 1.73 | Nobox | NOBOX oogenesis homeobox |
| 1.73 | Speer8-ps1 | spermatogenesis associated glutamate (E)-rich protein 8, pseudogene 1 |
| 1.72 | Syne2 | synaptic nuclear envelope 2 |
| 1.72 | Dusp1 | dual specificity phosphatase 1 |
| 1.71 | Tmc3 | transmembrane channel-like gene family 3 |
| 1.71 | Tnfaip3 | tumor necrosis factor, alpha-induced protein 3 |
| 1.68 | Hmgcs1 | 3-hydroxy-3-methylglutaryl-Coenzyme A synthase 1 |
| 1.65 | Insig1 | insulin induced gene 1 |
| 1.65 | Meig1 | meiosis expressed gene 1 |
| 1.64 | Ptch1 | patched homolog 1 |
| 1.63 | Fbxo47 | F-box protein 47 |
| 1.62 | Egr4 | early growth response 4 |
| 1.61 | Sqle | squalene epoxidase |
| 1.60 | Hnrnph3 | heterogeneous nuclear ribonucleoprotein H3 |
| 1.59 | Zfp872 | zinc finger protein 872 |
| 1.59 | Rsad2 | radical S-adenosyl methionine domain containing 2 |
| 1.58 | Pramef12 | PRAME family member 12 |
| 1.58 | Sertad4 | SERTA domain containing 4 |
| 1.57 | Sgk1 | serum/glucocorticoid regulated kinase 1 |
| 1.56 | Nefm | neurofilament, medium polypeptide |
| 1.56 | Lss | lanosterol synthase |
| 1.56 | Efhb | EF hand domain family, member B |
| 1.55 | Dhcr7 | 7-dehydrocholesterol reductase |
| 1.55 | Dusp18 | dual specificity phosphatase 18 |
| 1.55 | 1700028J19Rik | RIKEN cDNA 1700028J19 gene |
| 1.54 | Dgke | diacylglycerol kinase, epsilon |
| 1.54 | Ppp1r15a | protein phosphatase 1, regulatory (inhibitor) subunit 15A |
| 1.53 | Chd7 | chromodomain helicase DNA binding protein 7 |
| 1.51 | 4930583H14Rik | RIKEN cDNA 4930583H14 gene |
| 1.51 | Tcerg1l | transcription elongation regulator 1-like |
| 1.51 | Rrad | Ras-related associated with diabetes |
| 1.51 | Trim12a | tripartite motif-containing 12A |
| 1.51 | Tbx20 | T-box 20 |
| 1.50 | Mt1 | metallothionein 1 |
