# Supplementary material for: ROS amplification drives mouse spermatogonial stem cell self-renewal
Source: Life Sci Alliance. 2019 Apr 2;2(2):e201900374. doi: 10.26508/lsa.201900374 (PMC6448598; doi:10.26508/lsa.201900374)
Supplement: Supplementary file 4 [file LSA-2019-00374_TableS4.docx]

**Table S4 : RNA sequence analysis**

** Top 50 downregulated genes in *Nox1* KD**

**Top 50 downregulated genes in *Bcl6b* KD**

**Top 50 downregulated genes in *Mapk7* KO**

** Top 50 downregulated genes in *Mapk14* KO**
