# Supplementary material for: ROS amplification drives mouse spermatogonial stem cell self-renewal
Source: Life Sci Alliance. 2019 Apr 2;2(2):e201900374. doi: 10.26508/lsa.201900374 (PMC6448598; doi:10.26508/lsa.201900374)
Supplement: Supplementary file 5 [file LSA-2019-00374_TableS5.docx]

**Table S5 Raw values for candidate transcription factors determined by microarray**

| gene_ name | Nox1  cont1 | Nox1  KD1 | Nox1  cont2 | Nox1  KD2 | ERK5 cont1 | ERK5 KO1 | ERK5  cont2 | ERK5  KO2 | Bcl6b  cont1 | Bcl6d  KD1 | Bcl6b  cont2 | Bcl6d  KD2 | p38  cont1 | p38  KO1 | p38_  cont2 | p38  KO2 |
| --- | --- | --- | --- | --- | --- | --- | --- | --- | --- | --- | --- | --- | --- | --- | --- | --- |
| Bcl6b | 2325.3242 | 1906.1516 | 2405.8218 | 2102.755 | 1566.298 | 2049.5737 | 1852.6166 | 1835.4426 | 2417.6333 | 1930.2329 | 2416.889 | 2108.329 | 1310.0583 | 1634.5327 | 1096.5376 | 1626.8158 |
| Dmrt1 | 1390.6499 | 1171.8649 | 1367.7557 | 1291.7316 | 1398.0781 | 1342.1075 | 1455.8823 | 1942.1553 | 1382.6984 | 1361.3558 | 1312.37 | 1467.933 | 1369.176 | 1046.9464 | 1480.2095 | 1186.1466 |
| Etv5 | 1492.9332 | 1258.8251 | 1346.6549 | 1330.1117 | 871.20624 | 880.18207 | 795.599 | 1002.0571 | 1677.3312 | 1599.0576 | 1342.3932 | 1313.4392 | 731.7574 | 781.4807 | 833.1914 | 921.0536 |
| Foxo1 | 965.7694 | 912.15106 | 970.62646 | 1049.9745 | 782.662 | 872.18756 | 869.56305 | 996.0751 | 844.4359 | 911.8348 | 905.8035 | 956.08575 | 747.7314 | 656.3073 | 764.9824 | 617.61584 |
| Id4 | 452.127 | 464.48572 | 446.20718 | 501.48483 | 250.54185 | 297.66867 | 266.5344 | 307.81784 | 429.90866 | 427.8263 | 418.82068 | 406.8852 | 252.67435 | 237.02383 | 273.90707 | 279.42383 |
| Myc | 284.2753 | 334.20627 | 250.07335 | 347.81326 | 235.91751 | 269.6882 | 217.57082 | 218.2557 | 259.6656 | 222.43213 | 221.32365 | 184.0882 | 119.388725 | 219.733 | 124.34529 | 255.71417 |
| Mycn | 246.94586 | 261.55847 | 254.91748 | 309.1104 | 306.17245 | 318.1843 | 285.99844 | 313.40274 | 243.72263 | 263.88718 | 219.90228 | 284.6766 | 442.85406 | 389.04703 | 468.2638 | 384.8046 |
| Neurog3 | 166.12604 | 113.54117 | 136.49545 | 113.35788 | 78.244865 | 80.610146 | 83.658585 | 84.90119 | 139.00073 | 141.14203 | 119.202225 | 124.516136 | 91.9658 | 89.647285 | 130.9506 | 119.4306 |
| Pax7 | 257.9322 | 214.24841 | 250.75179 | 232.11209 | 187.78922 | 153.17616 | 157.4104 | 169.91174 | 259.32666 | 238.6552 | 211.73622 | 202.5429 | 150.2062 | 159.05244 | 167.025 | 182.79773 |
| Pou2f1 | 789.944 | 673.7179 | 860.10913 | 783.5275 | 749.776 | 782.5063 | 755.3187 | 729.38184 | 717.5541 | 751.1848 | 762.7662 | 744.82227 | 701.5977 | 674.51556 | 735.21735 | 712.7673 |
| Pou3f1 | 981.55206 | 623.786 | 809.5832 | 826.9029 | 623.2316 | 562.5675 | 546.23035 | 748.6221 | 836.45624 | 669.85596 | 867.97516 | 754.9219 | 612.0685 | 627.8405 | 687.7108 | 592.00946 |
| Pou5f1 | 320.45956 | 305.71902 | 279.5101 | 302.9833 | 295.5183 | 330.02908 | 318.88968 | 285.17175 | 326.51797 | 340.26193 | 258.22803 | 293.94196 | 175.903 | 163.4104 | 169.83926 | 190.01376 |
| Sohlh1 | 395.551 | 428.94275 | 374.44766 | 441.48645 | 414.1599 | 451.47327 | 434.59244 | 359.9861 | 402.41708 | 378.60678 | 358.69534 | 407.12735 | 337.07437 | 287.48694 | 405.1873 | 315.12112 |
| Sohlh2 | 823.21497 | 929.2625 | 798.94293 | 736.7562 | 712.3184 | 759.94226 | 623.41376 | 665.4974 | 726.83295 | 742.90155 | 862.3955 | 1117.5963 | 1001.89417 | 830.93634 | 763.55115 | 769.90173 |
| Sox3 | 506.60495 | 420.63217 | 431.9455 | 454.45374 | 320.66086 | 328.347 | 383.85315 | 372.30014 | 430.79117 | 409.52298 | 415.97574 | 380.6308 | 351.87158 | 309.48557 | 429.0902 | 382.01263 |
| Stat3 | 1585.1058 | 1475.8247 | 1591.5135 | 1706.752 | 1350.3774 | 1596.1873 | 1651.0259 | 1834.6614 | 1558.2942 | 1216.5762 | 1468.7036 | 1252.4819 | 1686.5151 | 1617.8065 | 1878.4092 | 1922.3003 |
| Taf4b | 1143.9628 | 1123.4673 | 1156.1078 | 1134.4337 | 1189.906 | 1185.4607 | 1345.1593 | 1471.9547 | 1000.77625 | 1110.0101 | 1148.5629 | 1315.3872 | 1013.30634 | 929.2146 | 1193.0312 | 1070.2416 |
| Tsc22d3 | 614.3214 | 618.60834 | 615.067 | 655.2336 | 887.50037 | 876.3903 | 735.04535 | 713.95795 | 609.229 | 599.0486 | 558.02704 | 549.6779 | 697.59235 | 699.3589 | 882.1042 | 266.957 |
