# Supplementary material for: ROS amplification drives mouse spermatogonial stem cell self-renewal
Source: Life Sci Alliance. 2019 Apr 2;2(2):e201900374. doi: 10.26508/lsa.201900374 (PMC6448598; doi:10.26508/lsa.201900374)
Supplement: Supplementary file 6 [file LSA-2019-00374_TableS6.docx]

**Table S6 Raw values for candidate transcription factors determined by RNA-seq**

| gene_ name | Nox1  cont1 | Nox1  KD1 | Nox1  cont2 | Nox1  KD2 | ERK5 cont1 | ERK5 KO1 | ERK5  cont2 | ERK5  KO2 | Bcl6b  cont1 | Bcl6d  KD1 | Bcl6b  cont2 | Bcl6d  KD2 | p38  cont1 | p38  KO1 | p38_  cont2 | p38  KO2 |
| --- | --- | --- | --- | --- | --- | --- | --- | --- | --- | --- | --- | --- | --- | --- | --- | --- |
| Bcl6b | 132.605 | 137.217 | 152.678 | 76.5041 | 124.421 | 125.345 | 108.824 | 112.46 | 113.228 | 105.017 | 172.375 | 115.786 | 110.476 | 104.323 | 161.67 | 165.475 |
| Dmrt1 | 162.961 | 196.275 | 176.573 | 158.626 | 205.7 | 237.795 | 241.512 | 264.787 | 142.071 | 151.657 | 153.502 | 193.921 | 215.876 | 239.012 | 180.609 | 176.106 |
| Etv5 | 177.62 | 201.609 | 188.436 | 102.91 | 84.7004 | 91.6582 | 90.5913 | 91.8757 | 123.913 | 109.564 | 153.718 | 160.277 | 54.9394 | 51.3083 | 56.5353 | 65.3424 |
| Foxo1 | 19.8957 | 21.3256 | 23.0884 | 27.8292 | 28.76 | 31.0761 | 27.0941 | 28.4679 | 21.4085 | 20.1945 | 19.6779 | 21.8513 | 26.0387 | 24.6102 | 34.4469 | 31.9344 |
| Id4 | 23.0097 | 23.8113 | 24.2253 | 7.99402 | 9.95133 | 10.6178 | 11.7955 | 13.3643 | 30.7978 | 26.758 | 24.0408 | 30.1208 | 11.6175 | 11.8502 | 11.3215 | 11.2051 |
| Myc | 9.329 | 3.53821 | 3.67435 | 1.36983 | 5.14243 | 3.61664 | 3.16098 | 2.6269 | 7.85776 | 8.04303 | 10.5214 | 7.05049 | 3.09947 | 3.58617 | 3.19016 | 1.86174 |
| Mycn | 36.9305 | 47.6224 | 35.2529 | 24.6625 | 42.195 | 43.3585 | 42.7676 | 44.2169 | 19.5069 | 20.971 | 38.9296 | 46.8124 | 41.4612 | 43.9506 | 27.1095 | 26.4189 |
| Neurog3 | 1.3894 | 1.82948 | 2.25543 | 2.00232 | 5.54843 | 5.65366 | 4.06205 | 3.11997 | 2.34441 | 1.58419 | 1.19158 | 1.08549 | 2.49096 | 3.38968 | 1.64416 | 2.08282 |
| Pax7 | 0.54793 | 2.03782 | 3.69954 | 1.43953 | 1.24894 | 1.78962 | 2.48678 | 2.7183 | 0.19984 | 0.31666 | 0.66925 | 1.05449 | 0.96841 | 0.89747 | 0.49943 | 0.39059 |
| Pou2f1 | 12.5932 | 15.0006 | 13.8637 | 13.7985 | 24.6625 | 21.4232 | 20.114 | 20.5113 | 5.63019 | 4.83727 | 13.8928 | 15.2009 | 11.8892 | 10.9624 | 26.7716 | 27.7384 |
| Pou3f1 | 37.3131 | 36.2099 | 37.8699 | 28.6968 | 35.3413 | 37.0383 | 34.0093 | 37.1637 | 38.3386 | 42.9317 | 47.5568 | 43.4786 | 55.6724 | 58.4004 | 65.5232 | 63.4434 |
| Pou5f1 | 140.367 | 159.889 | 135.027 | 64.6749 | 115.703 | 114.574 | 127.368 | 131.586 | 99.7882 | 95.4746 | 151.203 | 155.44 | 35.246 | 32.5576 | 22.6325 | 19.7246 |
| Sohlh1 | 23.1799 | 30.6845 | 74.9341 | 147.77 | 126.618 | 140.541 | 121.68 | 127.169 | 26.6367 | 30.4413 | 27.7802 | 21.0747 | 97.0318 | 112.16 | 102.773 | 100.863 |
| Sohlh2 | 109.365 | 146.658 | 108.037 | 90.841 | 79.9776 | 88.6493 | 91.3417 | 98.5788 | 97.5938 | 101.827 | 107.775 | 128.489 | 98.8723 | 105.264 | 92.8769 | 104.54 |
| Sox3 | 16.1271 | 22.2626 | 20.3352 | 30.924 | 31.1192 | 37.7854 | 39.0634 | 35.4414 | 22.5773 | 34.6548 | 12.4478 | 19.166 | 33.8783 | 43.124 | 47.9471 | 40.7365 |
| Stat3 | 129.053 | 142.791 | 120.057 | 176.535 | 163.662 | 144.426 | 161.833 | 149.572 | 92.3066 | 76.6781 | 160.322 | 112.099 | 114.739 | 84.346 | 156.039 | 166.164 |
| Taf4b | 39.095 | 40.0301 | 34.8973 | 33.7176 | 45.2849 | 44.1629 | 35.727 | 36.685 | 40.2976 | 38.1107 | 36.5632 | 36.7894 | 33.5799 | 31.5146 | 41.378 | 43.0282 |
| Tsc22d3 | 253.711 | 265.66 | 200.749 | 174.608 | 198.201 | 201.729 | 179.038 | 189.018 | 290.722 | 303.501 | 234.487 | 275.667 | 267.232 | 273.088 | 246.189 | 266.957 |
