# Supplementary material for: ROS amplification drives mouse spermatogonial stem cell self-renewal
Source: Life Sci Alliance. 2019 Apr 2;2(2):e201900374. doi: 10.26508/lsa.201900374 (PMC6448598; doi:10.26508/lsa.201900374)
Supplement: Supplementary file 7 [file LSA-2019-00374_TableS7.docx]

**Table S7: Chemicals used in this study**

| Name of chemical | Vendor | Final concentration |
| --- | --- | --- |
| A83-01 | Tocris, Minneapolis, MN | 100 nM |
| Apocynin | Tokyo Chemical Industry, TOKYO, JAPAN | 1 mM |
| BayK8644 | ApexBio, Houston, TX | 2 µM |
| BIRB796 | Selleck Chemicals, Houston, TX | 2 µM |
| BIX01294 | Sigma-Aldrich, St. Louis, MO | 1 µg/ml |
| BIX02189 | Medchem express, Monmouth Junction, NJ | 5 µM |
| Blebbistatin | Sigma-Aldrich, St. Louis, MO | 20 µM |
| Cx-4945 | Selleck Chemicals, Houston, TX | 10 µM |
| DPI | Sigma-Aldrich, St. Louis, MO | 1 µM |
| Forskolin | Selleck Chemicals, Houston, TX | 5 mM |
| Gö-6983 | Tocris, Minneapolis, MN | 5 µM |
| Kenpaullone | Wako Pure Chemical Industries, Tokyo, Japan | 5 µM |
| LPA | Sigma-Aldrich, St. Louis, MO | 2 mM |
| MHY1485 | Calbiochem, San Diego, CA | 2 µM |
| ML141 | Tocris, Minneapolis, MN | 10 µM |
| NaB | Wako Pure Chemical Industries, Tokyo, Japan | 250 µM |
| NAC | Sigma-Aldrich, St. Louis, MO | 0.5 mM |
| NSC87877 | Tocris, Minneapolis, MN | 50 µM |
| PMA | Wako Pure Chemical Industries, Tokyo, Japan | 10 nM |
| Pyrintegrin | Stemgent, Cambridge, MA | 2 µM |
| Quercetin | Cayman, Ann Arbor, MI | 1 µM |
| Repsox | Sigma-Aldrich, St. Louis, MO | 25 µM |
| SB203589 | Selleck Chemicals, Houston, TX | 30 µM |
| SC79 | Calbiochem, San Diego, CA | 5 µg/ml |
| Thiazovivin | Stemgent, Cambridge, MA | 0.5 µM |
| VX-745 | ApexBio, Houston, TX | 25 mM |
| XMD 8-92 | Tocris, Minneapolis, MN | 5 µM |
| Y27632 | Wako Pure Chemical Industries, Tokyo, Japan | 10 µM |
