# Supplementary material for: ROS amplification drives mouse spermatogonial stem cell self-renewal
Source: Life Sci Alliance. 2019 Apr 2;2(2):e201900374. doi: 10.26508/lsa.201900374 (PMC6448598; doi:10.26508/lsa.201900374)
Supplement: Supplementary file 8 [file LSA-2019-00374_TableS8.docx]

**Table S8: KD vectors used in this study**

| Target  gene | Target sequence |
| --- | --- |
| *Bcl6b* | TRCN0000084593, TRCN0000084594, TRCN0000084596, TRCN0000084597 |
| *Etv5* | TRCN0000054783, TRCN0000054784, TRCN0000054785, TRCN0000054786  TRCN0000054787 |
| *Nox1* | TRCN0000076603, TRCN0000076604, TRCN0000076605, TRCN0000076606  TRCN0000076607 |
| *Noxa2* | TRCN0000070658, TRCN0000070659, TRCN0000070660, TRCN0000070661  TRCN0000070662 |
| *Sohlh1* | TRCN0000198508, TRCN0000181617, TRCN0000181367, TRCN0000181285  TRCN0000198611 |
