# Supplementary material for: ROS amplification drives mouse spermatogonial stem cell self-renewal
Source: Life Sci Alliance. 2019 Apr 2;2(2):e201900374. doi: 10.26508/lsa.201900374 (PMC6448598; doi:10.26508/lsa.201900374)
Supplement: Supplementary file 9 [file LSA-2019-00374_TableS9.docx]

**Table S9: Antibodies used in this study**

| Name of antigen | Vendor |
| --- | --- |
| Alexa fluor 488 anti-Rabbit IgG (H+L) | Molecular Probes, Carlsbad, CA (A21206) |
| Alexa fluor 555 anti-Goat IgG | Molecular Probes, Carlsbad, CA (A21432) |
| Alexa fluor 647 anti-Rabbit IgG (H+L) | Molecular Probes, Carlsbad, CA (A31573) |
| Alexa fluor 647 anti-Goat IgG | Molecular Probes, Carlsbad, CA (A21447) |
| Goat anti-human BCL6B | Santa Cruz, Dallas, TX (sc-107454) |
| Goat anti-mouse GATA4 | Santa Cruz, Dallas, TX (sc-1237) |
| Goat anti-rat GFRA1 | R & D systems, Minneapolis, MN (AF560) |
| HRP-anti-mouse IgG | Cell signaling, Danvers, MA (#7076) |
| HRP-anti-rabbit IgG | Cell signaling, Danvers, MA (#7074) |
| HRP-anti-rat IgG | DakoCytomation, Carpinteria,CA (P0162) |
| Mouse anti-mouse ACTB | Sigma-Aldrich, St. Louis, MO (A5441) |
| Rabbit anti-human BCL6B | Thermo Fisher Scientific, Waltham, MA  (PA5-41693) |
| Rabbit anti-human phospho MAPK14 (Thr180/Tyr182) | Cell signaling, Danvers, MA (#4511) |
| Rabbit anti-human phospho MAPK7 (Thr218/Tyr220) | Cell signaling, Danvers, MA (#3371) |
| Rabbit anti-human phospho Map2k5 (Ser311/Thr315) | abcam, Cambridge (ab70608) |
