# Supplementary material for: ROS amplification drives mouse spermatogonial stem cell self-renewal
Source: Life Sci Alliance. 2019 Apr 2;2(2):e201900374. doi: 10.26508/lsa.201900374 (PMC6448598; doi:10.26508/lsa.201900374)
Supplement: Supplementary file 10 [file LSA-2019-00374_TableS10.docx]

**Table S10: PCR Primers used in this study**

|  | Forward | Reverse |
| --- | --- | --- |
| *Bcl6b* | AAGCCATACCACTGTGACCC | ATCAAGCCTGAGCCTACAGC |
| *Cyba* | TGGACGTTTCACACAGTGGT | CCGAAAAGCTTCACCACAGA |
| *Dmrt1* | ATGAAGACCTCAGAGAGCCG | CAAGCCAGAATCTTGACTGC |
| *Etv5* | AACTTGGTGCTTCATGCTCC | ACTTAGCACCAAGAGCCTGC |
| *Foxo1* | GTGAAGAGCGTGCCCTACTT | TCCTTCATTCTGCACTCGAA |
| *Hprt* | GCTGGTGAAAAGGACCTCT | CACAGGACTAGAACACCTGC |
| *Id4* | GTTCACGAGCATTCACCGTA | AAGGTTGGATTCACGATTGC |
| *Mapk7*  *Exon4* | TGCTTTGGTATTGTCGGAGA | GTGGCTAGTGAGCCTGTGTG |
| *Mapk7*  *Exon1* | ATGCCCTTTTCCTGCTCCCCA | ATGAGGTCCAGTACCACATAG |
| *Mapk11* | CAGAAGGACCTCAGCAGTGTCT | GTACTGGCTGAAGTATGCGTGG |
| *Mapk12* | GGCTACTGGATGTGTTCACACC | CTGGATTCTGTCTTCACTCAGGG |
| *Mapk13* | CAGCGAGGATAAGGTCCAGTAC | GCTCACAGTCTTCATTCACAGCC |
| *Mapk14* | CCGAACGATACCAGAACCTGTC | ACGCAACTCTCGGTAGGTCCTT |
| *Mapk14*  *Exon2* | GGTCAGCAGCCTCGATGCAC | GACTGCCCCTCCAACCGTTC |
| *Mapk14*  *Exon12* | GCCCTCCCTCACTTCAGGAG | TGTGCTCGGCACTGGAGACC |
| *Myc* | TGCAGGACCTCACCGC | CTTCTTGCTCTTCTTCAGAGTCG |
| *Mycn* | CCGGAGAGGATACCTTGAGC | CCAGAGCGGAGGTCTTGG |
| *Neurog3* | GCCTCATTGGAGGAATTCC | AGATGCTTGAGAGCCTCCAC |
| *Nox1* | ACAGAGGAGAGCTTGGGTGA | CACTCCAGGAAGGAAATGGA |
| *Noxo1* | ACATTTGCCTTCTCCGTGTG | ACGTGTCAGCAATGGAGCAT |
| *Noxo2* | GGACACCTTCATTCGCCATA | GGGAACATCTCCTTCAGCAT |
| *Noxa1* | GCATTTGACCAAGCAGTGAC | CCAGGCTTGCAATTTGAAGT |
| *Noxa2* | AACATAGGCTGCGTGAACAC | GCCCCAGGATCTTGTAGTCT |
| *Pax7* | GACTCCGGATGTGGAGAAAA | CACTCGGCTAATCGAACTCA |
| *Pou2f1* | AGTCTTGCTGCTATGGCTGC | CGTTGCCAGTGTACTGTTGC |
| *Pou3f1* | TCTACGGTAACGTGTTCTCGC | ATCTTGTCCAGGTTGGTGG |
| *Pou5f1* | GGTTCTCATTGTTGTCGGCT | AGCTGCTGAAGCAGAAGAGG |
| *Rac1* | AGCTGTTGGTAAAACCTGCC | GGGACGCAATCTGTCATAAT |
| *Sohlh1* | ACCCTAGCTGGGAGCAACTCT | CCCGAGACACAGCAGATGGT |
| *Sohlh2* | GGATTAAAGGCCCCGTTGTC | ATCGCTCTTCCTCCCCTTGA |
| *Sox3* | TTCGCACTCGCAGCGCGCGTGCCTCGG | ACGGCCAAACTTTCGGTCCTC |
| *Stat3* | CCAAGTTCATCTGTGTGACACC | TGTCTCAAGTCACGTCTCTGC |
| *Taf4b* | TCACAAGAATCTGCCTCAGG | GCCACAAAGACAAGACGTAGC |
| *Tsc22d3* | CCCTAGACAACAAGATTGAGC | CTTCTCAAGCAGCTCACGAA |
